# Supplementary material for: Precise immunofluorescence canceling for highly multiplexed imaging to capture specific cell states
Source: Nat Commun. 2024 May 8;15:3657. doi: 10.1038/s41467-024-47989-9 (PMC11078938; doi:10.1038/s41467-024-47989-9)
Supplement: Supplementary file 1 — Supplementary Information [file 41467_2024_47989_MOESM1_ESM.pdf]

Supplementary Information

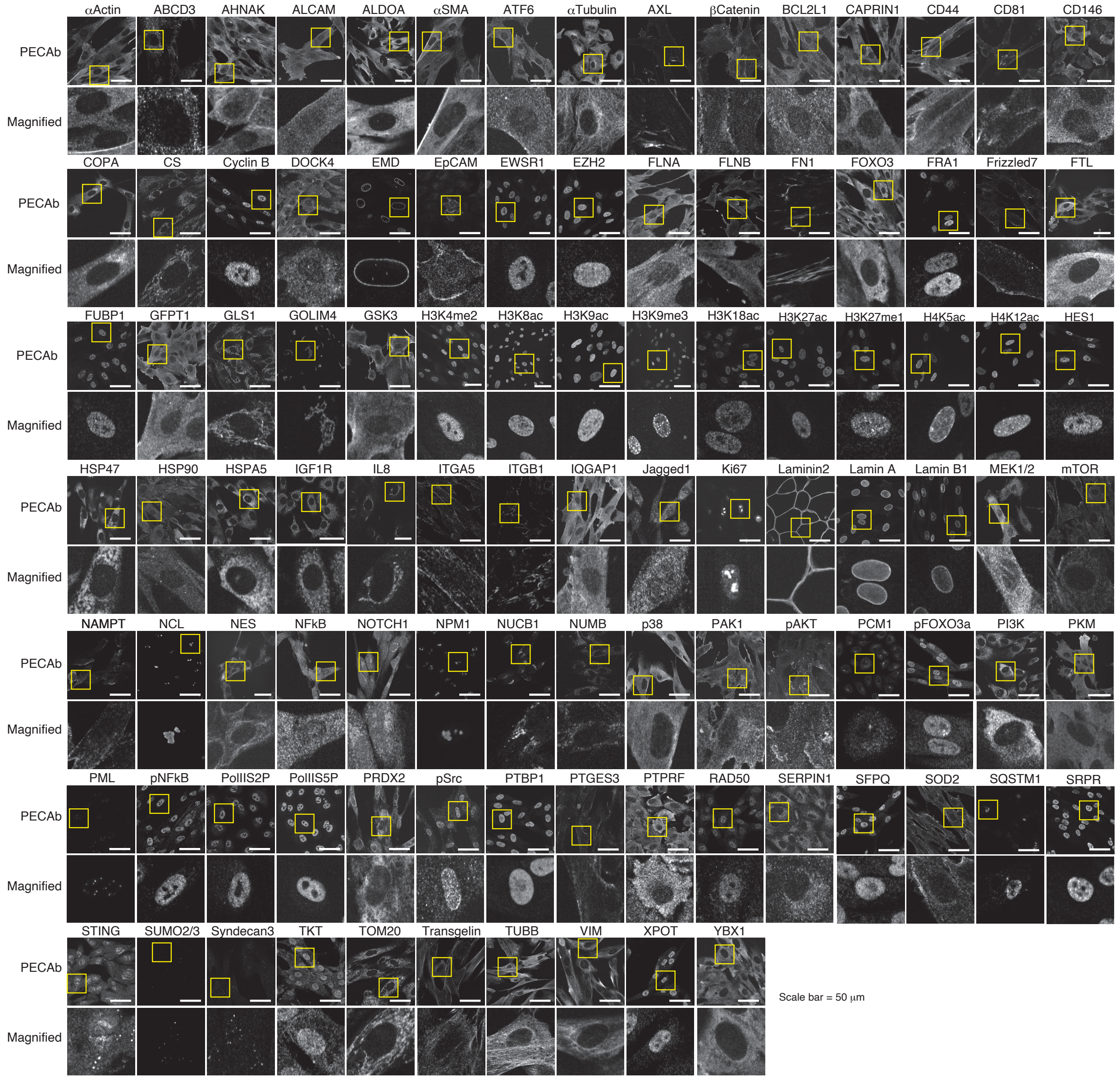

Supplementary Fig. 1. Evaluation of PECAbs. Samples (IMR90, A549, and mouse TA tissue) were stained with PECAbs. Images were obtained by using a 63 × oil objective lens (Plan Apo 1.40 NA). Regions in yellow square are magnified and indicated below of for each image. Scale bars = 50 μm. Experiments were performed at least 2 times and representative images are shown.

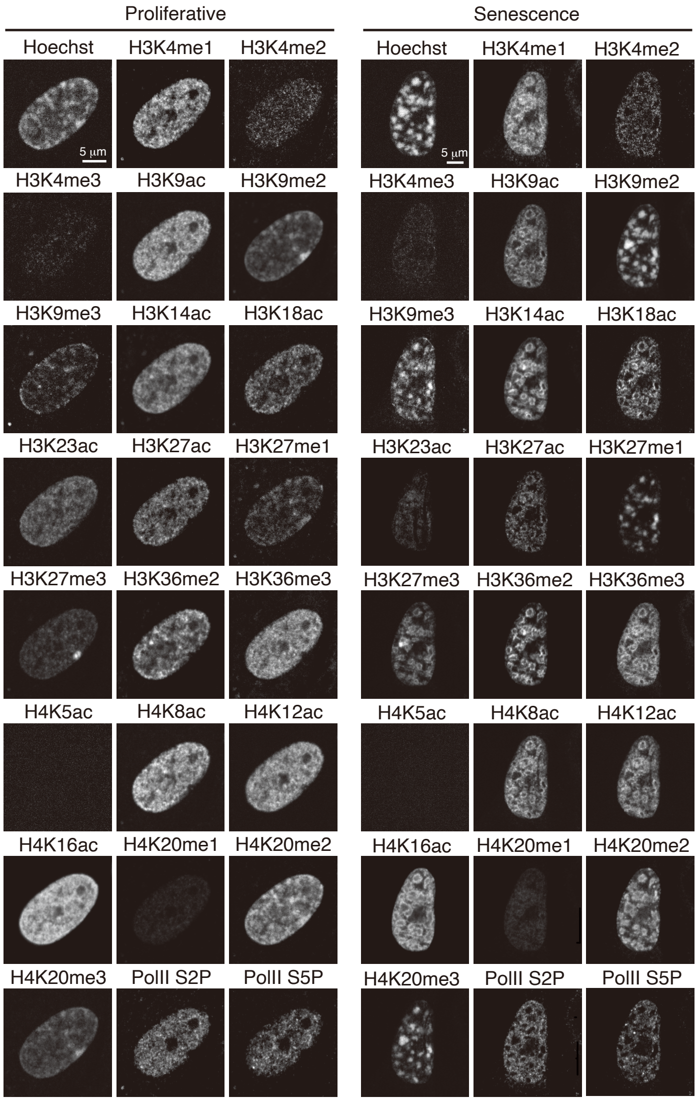

Supplementary Fig. 2. Multiplexed imaging of histone modifications using PECAb. Representative images of IMR90 ER:Ras cells stained with indiated PECAbs. Scale bar = 5 μm. Experiments were performed for 2 times and representative images are shown.

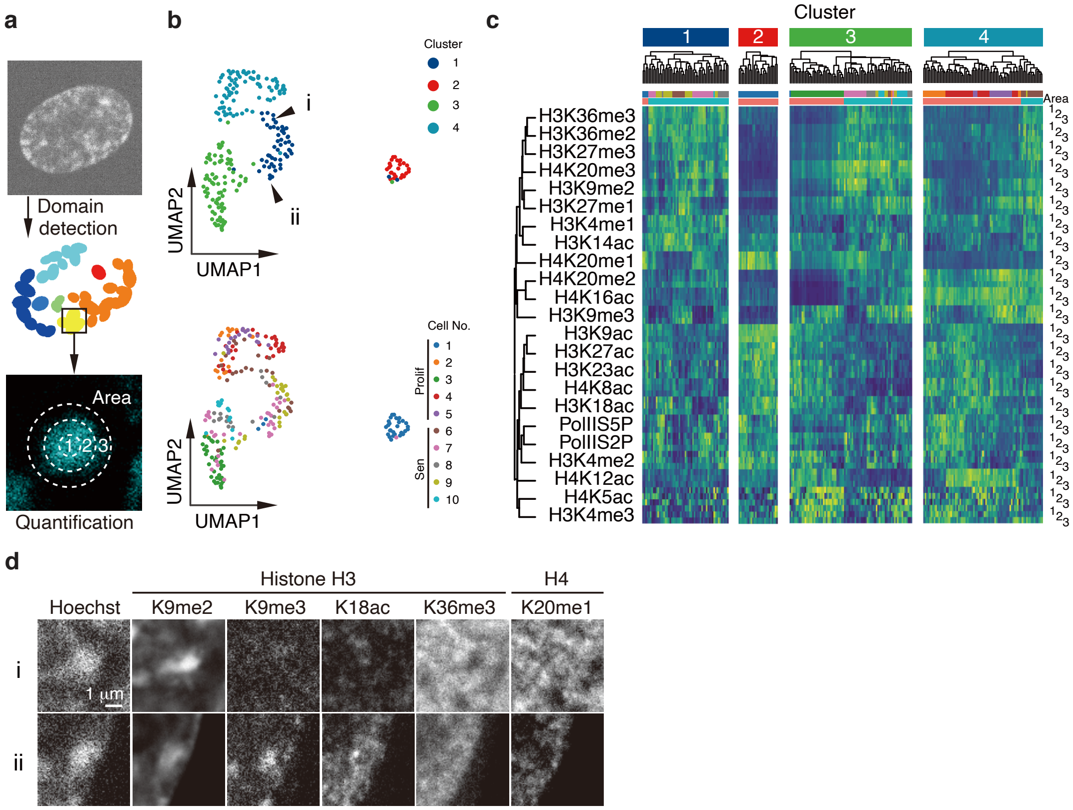

Supplementary Fig. 3. Quantitative analysis of the multiplexed imaging data in Supplementary Fig. 2. a. Quantification of the immunofluorescence signals. Hoechst-dense chromatin domains were detected and separated into three regions then, fluorescence intensities were counted. **b.** UMAP visualization colored by Leiden clusters (top) and original cells (bottom). **c.** Heatmap showing the quantitative value of clustered domains indicated in the UMAP. **d.** Representative images of the chromatin domains indicated in the UMAP (Supplementary Fig. 3b). Scale bar = 1 μm. Experiments were performed for 2 times and representative images are shown.

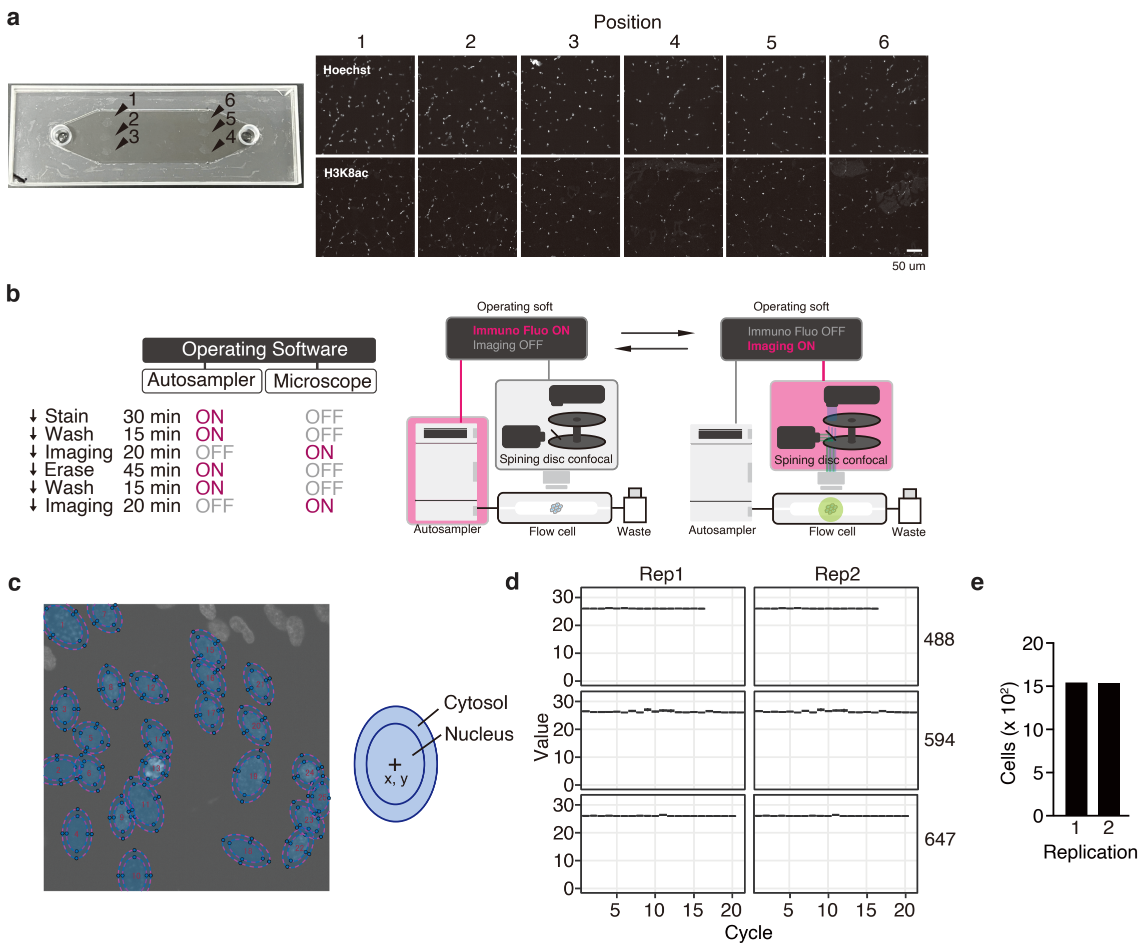

Supplementary Fig. 4. Establishment of the experimental system for SeqIS. a. Assessment of position effect for PECAb staining in the microfluidics chip. Mouse tibialis anterior (TA) tissue slices were placed on the arrowhead positions (left). Representative images of mouse TA stained with anti H3K8ac PECAb (Right). **b.** Schematics of the automatic device. **c.** Representative image of ellipse fitting. Hoechst images were used to detect nuclei and their locations (left panel). The ellipse was used to approximate the analysis area of the cell (right panel). **d.** Quantification of erased signals after TCEP treatment of samples. **e.** Cell numbers obtained in the experiments. All experiments were performed at least 2 times.

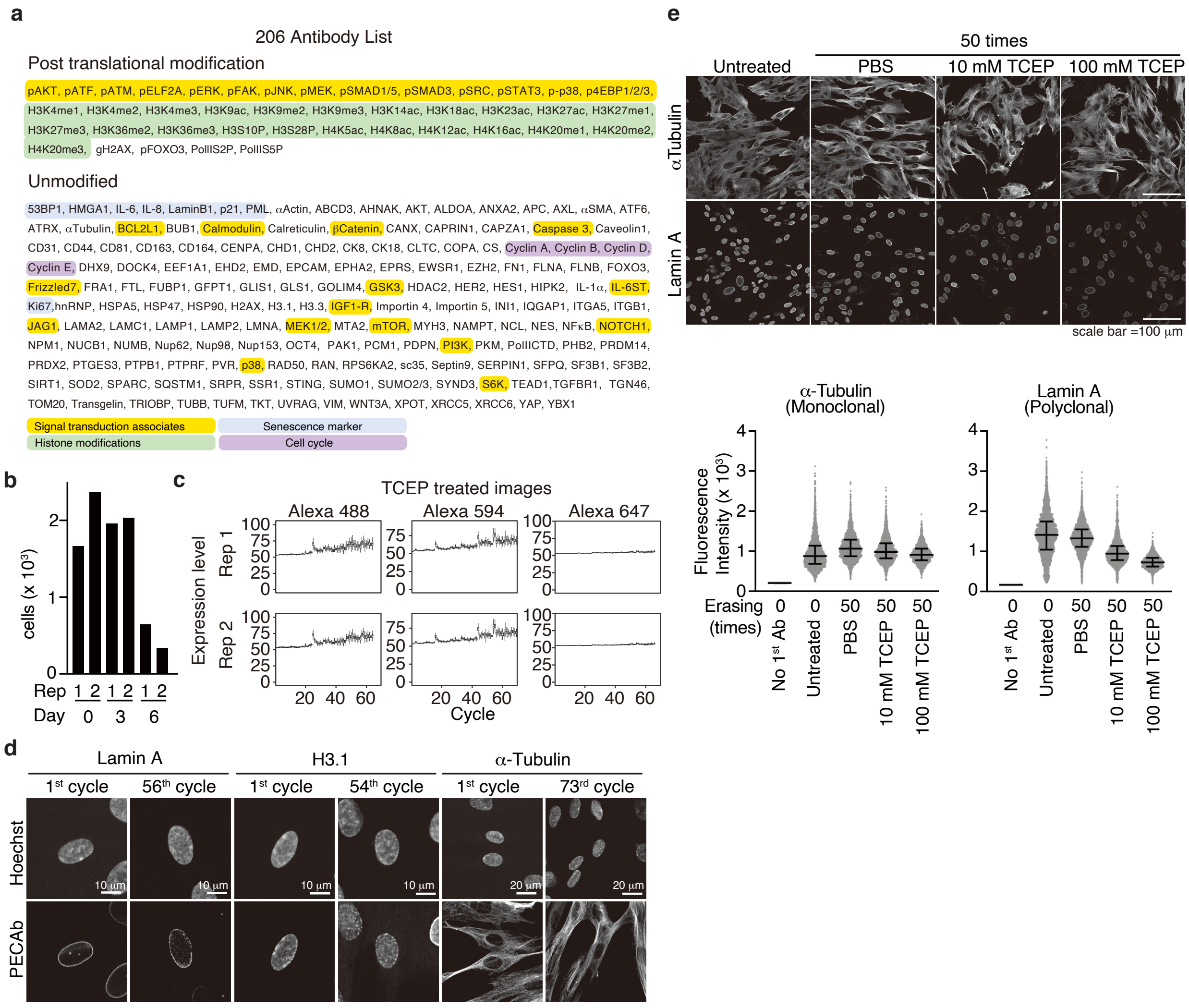

Supplementary Fig. 5. Evaluation of multiplexed imaging data in Fig 3. a. Antibodies used in the experiment for Fig.3. **b.** Detected cell numbers in the experiment. **c.** Quantified retained signals. Images of TCEP-treated cells were acquired and quantified values were plotted for each cycle. **d.** Comparison of PECAb staining patterns of IMR90:ER-ras cells at the indicated SeqIS cycle performed in Fig.3. The 1st cycle and indicated latter cycle images were obtained from separate specimens. Scale bars = 10 µm for Lamin A and H3.1, 20 µm for α-Tubulin. **e.** Top panel: fixed IMR90 cells were incubated with indicated 1st antibodies and then captured with specific 2nd antibodies. Bottom panel: fluorescence intensity of staining was measured. n = 7468, 3875, 3036, 2611, 2824 cells from two biological replicates of No 1st Ab, Untreated, PBS × 50 times, 10 mM TCEP × 50 times, and 100 mM TCEP × 50 times, respectively.

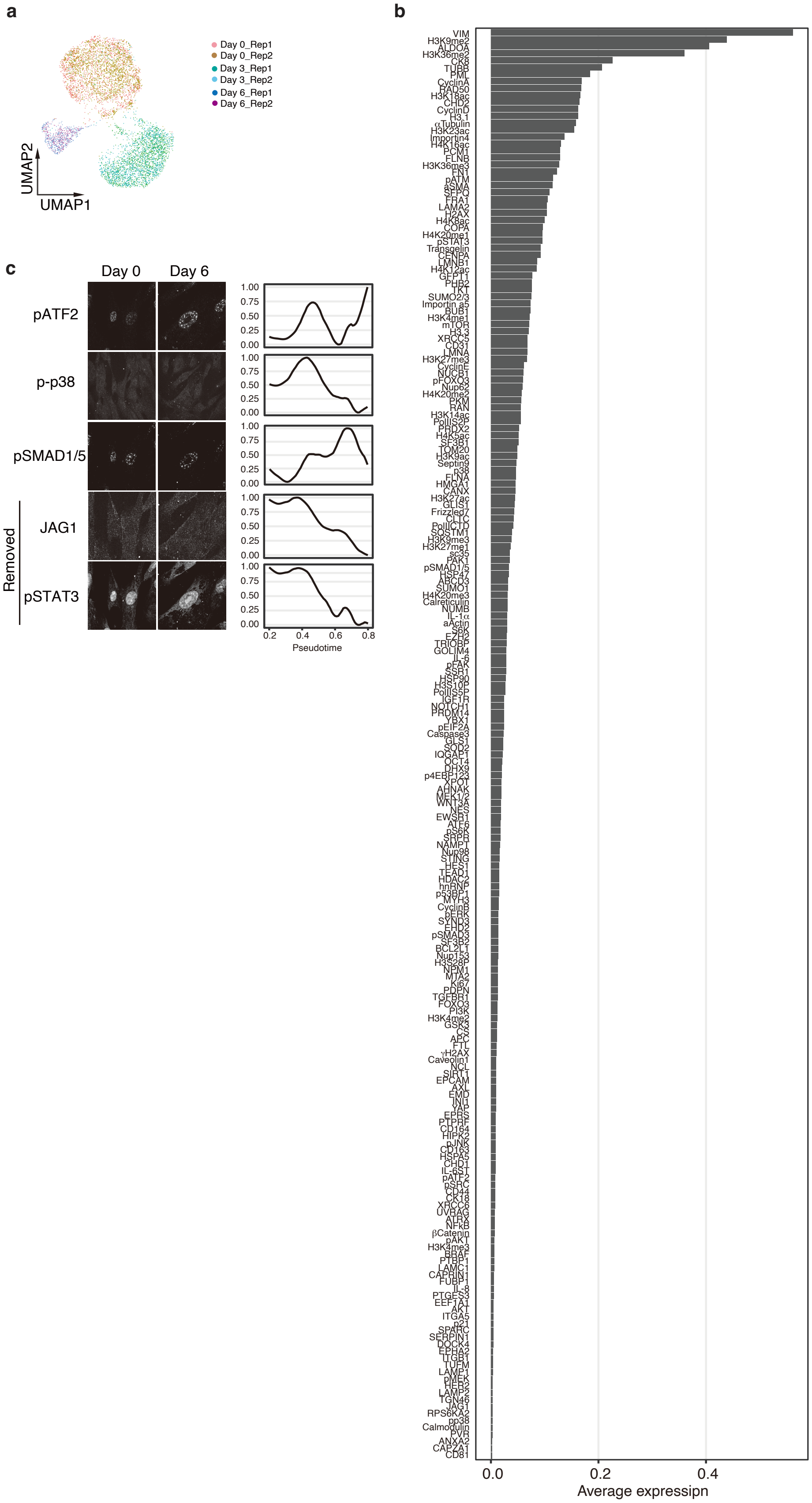

a
Day 0_Rep1
Day 0_Rep2
Day 3_Rep1
Day 3_Rep2
Day 6_Rep1
Day 6_Rep2
UMAP2
UMAP1
b
VIM
0.0
0.2
0.4
Average expressipn
c
Day 0
Day 6
pATF2
p-p38
pSMAD1/5
JAG1
pSTAT3
Removed
1.00
0.75
0.50
0.25
0.00
0.2
0.4
0.6
0.8
Pseudotime

Supplementary Fig. 6. Evaluation of quantified dataset in Fig.3. a. UMAP projection indicating reproducibility of experiments. **b.** Average expression values of quantified data (n = 6,693 cells). **c.** Representative immunofluorescent images of IMR90 cells. Fold changes in low-to-low expression stretched by scaling (e.g. JAG1 and pSTAT3) were removed by visual inspection. All data were from biologically duplicated experiments shown in Fig.3.

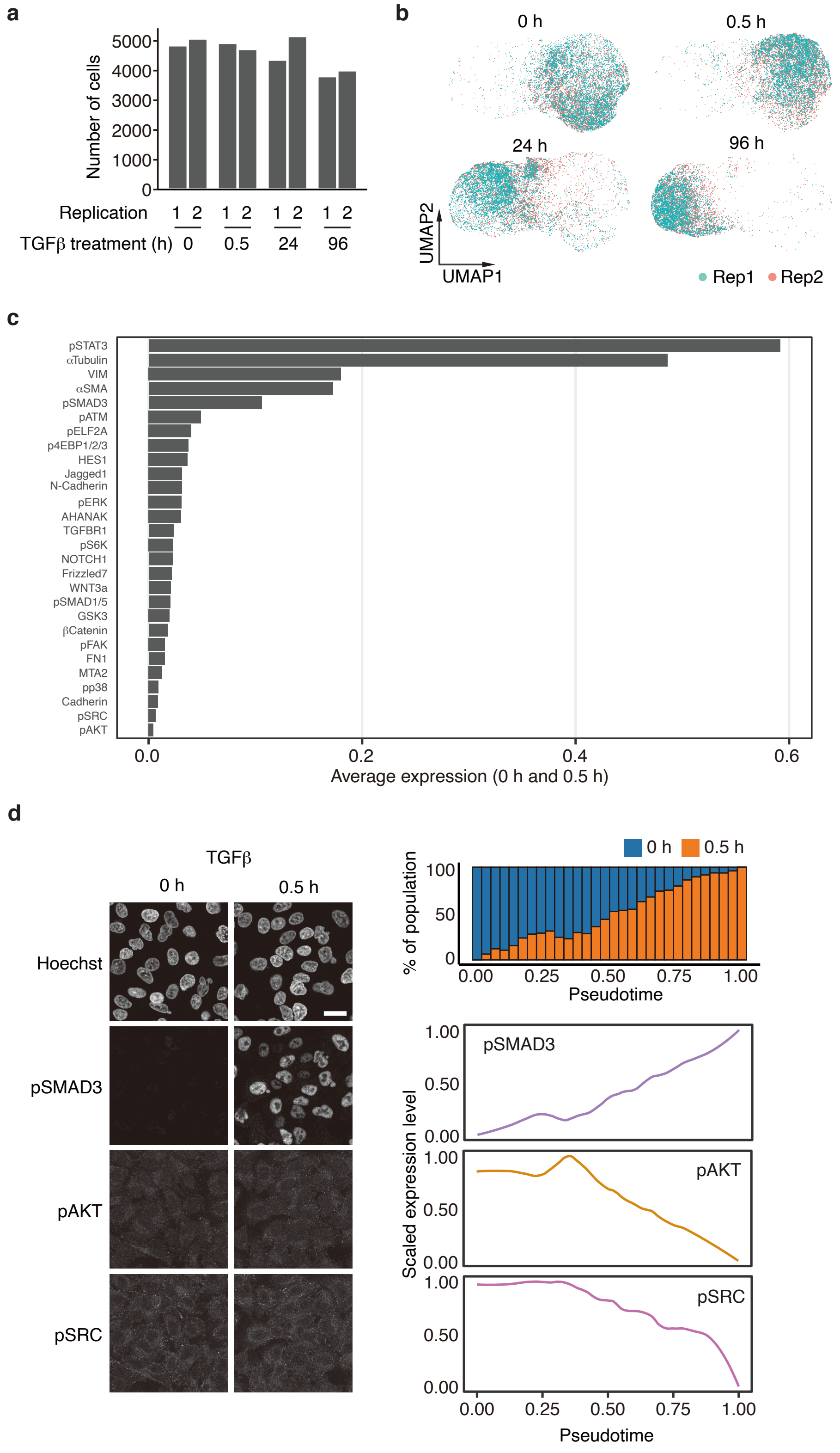

a
Number of cells
5000
4000
3000
2000
1000
0
Replication
1 2
TGFβ treatment (h)
0
0.5
24
96
b
0 h
0.5 h
24 h
96 h
UMAP2
UMAP1
Rep1
Rep2
c
pSTAT3
αTubulin
VIM
αSMA
pSMAD3
pATM
pELF2A
p4EBP1/2/3
HES1
Jagged1
N-Cadherin
pERK
AHANAK
TGFBR1
pS6K
NOTCH1
Frizzled7
WNT3a
pSMAD1/5
GSK3
βCatenin
pFAK
FN1
MTA2
pp38
Cadherin
pSRC
pAKT
0.0
0.2
0.4
0.6
Average expression (0 h and 0.5 h)
d
TGFβ
0 h
0.5 h
Hoechst
pSMAD3
pAKT
pSRC
0 h
0.5 h
% of population
100
50
0
0.00
0.25
0.50
0.75
1.00
Pseudotime
Scaled expression level
pSMAD3
pAKT
pSRC
0.00
0.50
1.00
Pseudotime

Supplementary Fig. 7. Evaluation of multiplexed imaging data showing in Fig.4. a. Detected cell numbers in the experiment. Experiments were performed for 2 times. **b.** UMAP projection indicating reproducibility of experiments. **c.** Average expression values of quantified data (n = 36,955 cells). **d.** Representative immunofluorescent images of A549 cells (left panel). Scale bar = 20 μm. Cell populations on the constructed pseudotime are shown in the right top panel. Fold changes in low-to-low expression stretched by scaling (right bottom panel) (e.g. pAKT and pSRC) were removed by visual inspection.

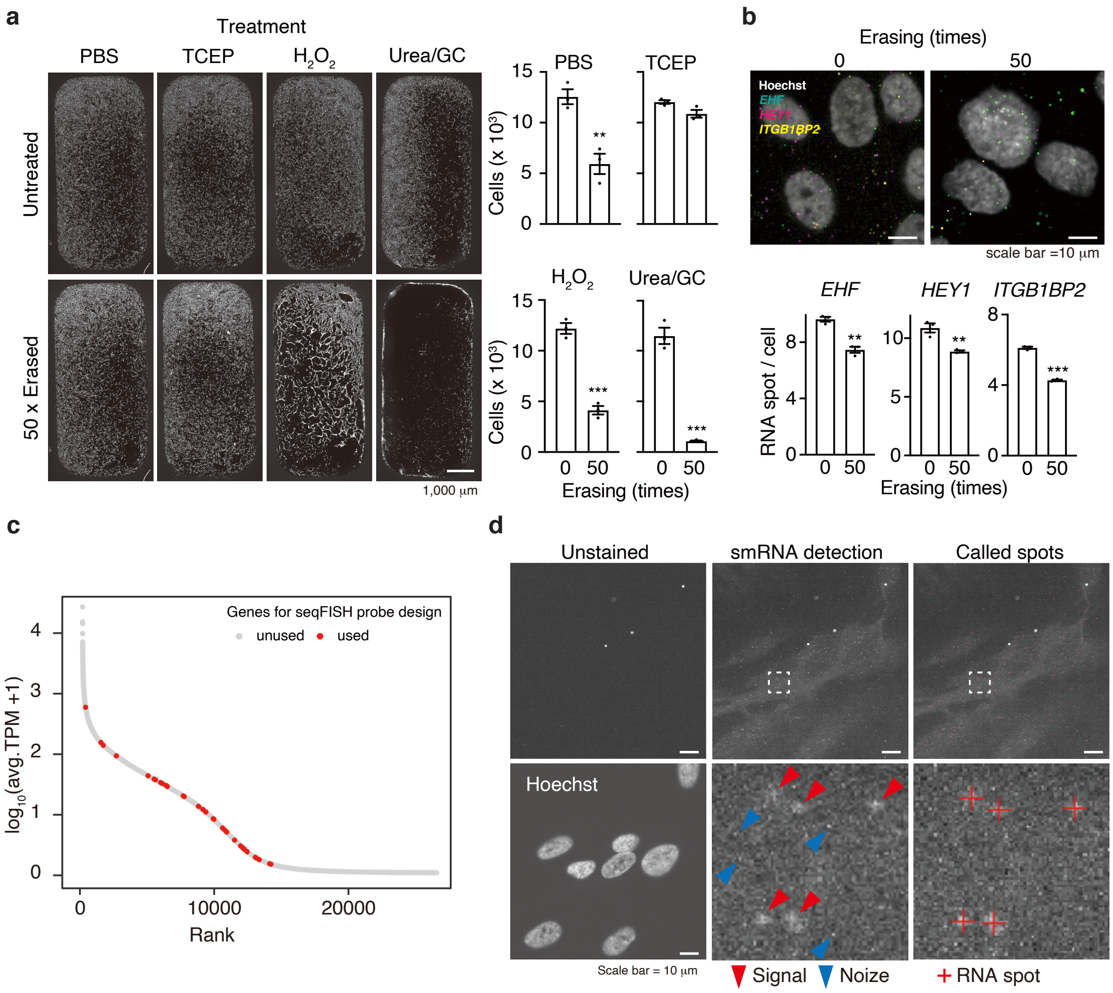

Supplementary Fig. 8. Evaluation of sample conditions for seqFISH. a. Fluorescence images of IMR90 cells. Fixed cells were treated 50 times with the indicated reagents and stained with Hoechst. Left panel, representative fluorescence images. Scale bar = 1,000 µm. Right panel, cell numbers in the images were counted after the indicated treatment. Values are the mean ± SEM. P values are: 0.006(PBS), 0.0504(TCEP), 0.0003(H_2O_2), and 0.0002(Urea/GC), $^{**}P < 0.01$, $^{***}P < 0.001$, two-tailed, unpaired Student's t-test (n = 3 biologically independent experiments). **b.** Seq-smFISH after erasing cycles. IMR90 cells were treated 50 times with TCEP and subjected to Seq-smFISH. Top panel, representative FISH images of IMR90 cells. Scale bar, 10 µm. Bottom panel, quantified RNA spots. Values are the mean ± SEM. P values are: 0.0014(*EHF*), 0.0075(*HEY1*), and <0.0001(*ITGB1BP1*), $^{**}P < 0.01$, $^{***}P < 0.001$, two-tailed, unpaired Student's t-test (n = 3 biologically independent experiments). **c.** Selected genes for seq-smFISH in Fig. 5. Genes expressed at high, medium, and low levels were selected from IMR90 cell RNA-seq data (GSE72404), indicated by red dots. **d.** Detection of specific RNA spots. Dotted square regions are magnified in the bottom panels. Red crosses indicate called RNA spots.

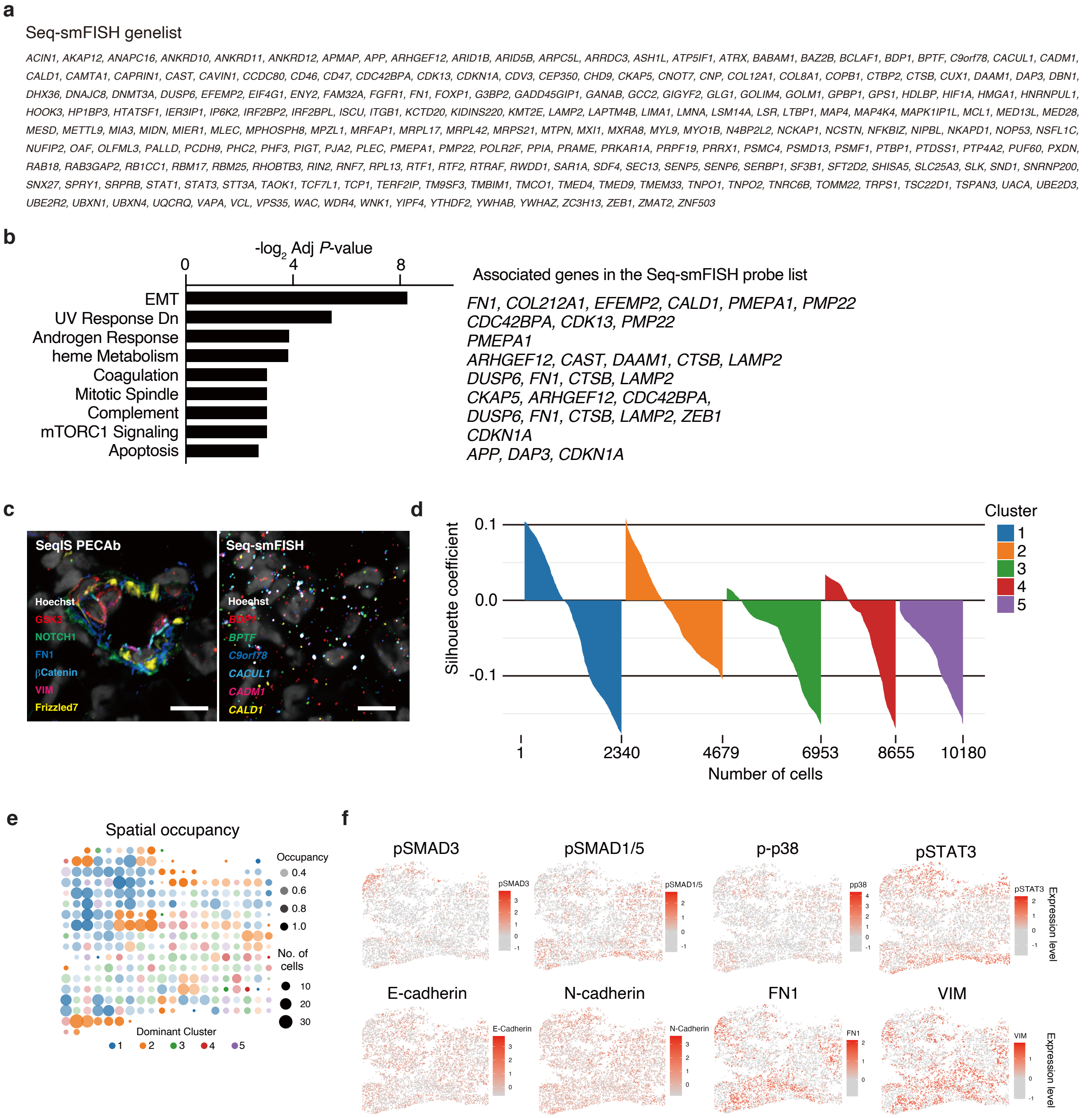

Supplementary Fig. 9. Analysis of spatial gene and protein expressions in uterine carcinosarcoma. a. Gene list used for the Seq-smFISH probe design. **b.** GSEA of genes used in the Seq-smFISH probe design. **c.** Representative images of SeqIS and Seq-smFISH of the same location. Scale bar = 10 μm. **d.** Silhouette coefficient of clustered cells to analyze spatial occupancy. A score > 0 indicates proximity to cells in the same cluster, and a score < 0 indicates proximity to cells in other clusters. **e.** Spatial occupancy of clustered cells in the tissue. **f.** Spatial expression of indicated proteins in the tissue.

Supplementary note

1. Quantitative analysis of chromatin domains.

We profiled the structures to confirm their applicability to high-resolution analysis (Supplementary Fig. 3). Selecting five nuclei each from proliferative and senescence IMR90 cells, we defined Hoechst-dense regions as domains divided into three layers, and quantified signals. The quantified results were projected on the uniform manifold approximation and projection (UMAP). Each point indicates a single domain. Domain profiles were grouped into four clusters, with Cluster 1 being enriched in chromatin domains derived from senescent cells. Variations were further observed within Cluster 1, and by comparing chromatin domain images with distant UMAP distances within Cluster 1, significant differences were observed, particularly in the aggregation of H3K9me3. These findings underscore the functionality of PECAb in analyzing microscales such as nuclear structure.
