# Supplementary material for: Precise immunofluorescence canceling for highly multiplexed imaging to capture specific cell states
Source: Nat Commun. 2024 May 8;15:3657. doi: 10.1038/s41467-024-47989-9 (PMC11078938; doi:10.1038/s41467-024-47989-9)
Supplement: Supplementary file 2 — Reporting Summary [file 41467_2024_47989_MOESM2_ESM.pdf]

nature portfolio

Corresponding author(s): Yasuyuki Ohkawa

Last updated by author(s): Apr 17, 2024

Reporting Summary

Nature Portfolio wishes to improve the reproducibility of the work that we publish. This form provides structure for consistency and transparency in reporting. For further information on Nature Portfolio policies, see our Editorial Policies and the Editorial Policy Checklist.

Statistics

For all statistical analyses, confirm that the following items are present in the figure legend, table legend, main text, or Methods section.

n/a	Confirmed	
☐	☒	The exact sample size (n) for each experimental group/condition, given as a discrete number and unit of measurement
☐	☒	A statement on whether measurements were taken from distinct samples or whether the same sample was measured repeatedly
☐	☒	The statistical test(s) used AND whether they are one- or two-sided<br>*Only common tests should be described solely by name; describe more complex techniques in the Methods section.*
☐	☒	A description of all covariates tested
☐	☒	A description of any assumptions or corrections, such as tests of normality and adjustment for multiple comparisons
☐	☒	A full description of the statistical parameters including central tendency (e.g. means) or other basic estimates (e.g. regression coefficient) AND variation (e.g. standard deviation) or associated estimates of uncertainty (e.g. confidence intervals)
☐	☒	For null hypothesis testing, the test statistic (e.g. F, t, r) with confidence intervals, effect sizes, degrees of freedom and P value noted<br>*Give P values as exact values whenever suitable.*
☒	☐	For Bayesian analysis, information on the choice of priors and Markov chain Monte Carlo settings
☒	☐	For hierarchical and complex designs, identification of the appropriate level for tests and full reporting of outcomes
☒	☐	Estimates of effect sizes (e.g. Cohen's d, Pearson's r), indicating how they were calculated

Our web collection on statistics for biologists contains articles on many of the points above.

Software and code

Policy information about availability of computer code

Data collection	Fusion software(ANDOR v2.3.0.44) was used to obtain fluorescence images.
Data analysis	Bowtie(v12.2.0), BLAST+(v2.2.31), Trim Galore(v0.6.10), HISAT2(v2.2.1), FeatureCounts(v2.0.4), UMI-tools(v1.1.4), R(v4.2.1), DESeq2(v1.34.0), uwot R package(v0.1.10), igraph R package(v1.5.0), Seurat (v4.3.0), phateR(v1.0.7), slingshot(v2.6.0), GraphPadPrism(v8.4.3), Matlab were used for data analysis. ChemDraw(v23.01) was used to draw chemical structures. All code for data analysis is available at https://github.com/tfwis/PECAb.

For manuscripts utilizing custom algorithms or software that are central to the research but not yet described in published literature, software must be made available to editors and reviewers. We strongly encourage code deposition in a community repository (e.g. GitHub). See the Nature Portfolio guidelines for submitting code & software for further information.

Data

Policy information about availability of data

All manuscripts must include a data availability statement. This statement should provide the following information, where applicable:

- Accession codes, unique identifiers, or web links for publicly available datasets
- A description of any restrictions on data availability
- For clinical datasets or third party data, please ensure that the statement adheres to our policy

The RNA-seq, scRNA-seq data in this study were deposited in the GSE242887 [https://www.ncbi.nlm.nih.gov/geo/query/acc.cgi?acc=GSE242887]. The source and processed data from this study are available in a Zenodo repository [https://doi.org/10.5281/zenodo.10655938] and 'Source Data' file. Molecular Signatures

Database (MSigDB) [https://www.gsea-msigdb.org/gsea/msigdb/] was used for Gene Set Enrichment Analysis. RefSeq database was used for [https://www.ncbi.nlm.nih.gov/refseq/].

Research involving human participants, their data, or biological material

Policy information about studies with human participants or human data. See also policy information about sex, gender (identity/presentation), and sexual orientation and race, ethnicity and racism.

Reporting on sex and gender	Uterus carcinosarcoma samples were obtained from female patients.
Reporting on race, ethnicity, or other socially relevant groupings	Not associated for this study.
Population characteristics	Not associated for this study.
Recruitment	Participants were not recruited. A part of surgically removed tissue from patients were used.
Ethics oversight	Ethical approval was obtained from the internal review boards of the Japanese Foundation for Cancer Research (IRB-ID: M22116-00) for experiments using human tissues.

Note that full information on the approval of the study protocol must also be provided in the manuscript.

Field-specific reporting

Please select the one below that is the best fit for your research. If you are not sure, read the appropriate sections before making your selection.

☒ Life sciences ☐ Behavioural & social sciences ☐ Ecological, evolutionary & environmental sciences

For a reference copy of the document with all sections, see nature.com/documents/nr-reporting-summary-flat.pdf

Life sciences study design

All studies must disclose on these points even when the disclosure is negative.

Sample size	Sample size design was not performed, since the objective was to demonstrate the technology.
Data exclusions	Image data from areas where samples were detached during sequential immuno-staining or FISH experiments were excluded from the analysis.
Replication	Spatial omics experiments were performed with 2 biological replicates per condition. Obtained dataset was highly reproducible. Another experiments were replicated as indicated in the legends, in order to allow statistical analysis of the results.
Randomization	N/A
Blinding	No blinding techniques were used.The investigators needed to know the treatment groups in order to perform the study.

Reporting for specific materials, systems and methods

We require information from authors about some types of materials, experimental systems and methods used in many studies. Here, indicate whether each material, system or method listed is relevant to your study. If you are not sure if a list item applies to your research, read the appropriate section before selecting a response.

Materials & experimental systems

n/a	Involved in the study	
☐	☒	Antibodies
☐	☒	Eukaryotic cell lines
☒	☐	Palaeontology and archaeology
☐	☒	Animals and other organisms
☒	☐	Clinical data
☒	☐	Dual use research of concern
☒	☐	Plants

Methods

n/a	Involved in the study	
☒	☐	ChIP-seq
☒	☐	Flow cytometry
☒	☐	MRI-based neuroimaging

Antibodies

Antibodies used	AHNAK (Abcam, ab68556, Clone# EM-09, RRID:AB_1209218), Annexin-2(Abcam, ab269511, Clone# D1/274.5), Axl (Abcam, ab89224,

Clone# MM0098-2N33, RRID:AB_2049189), Calnexin (Abcam, ab22595, RRID:AB_2069006), Calreticulin (Abcam, ab22683, Clone# FMC 75, RRID:AB_447253), Cleaved Caspase-3 (Abcam Cat# ab32042, Clone# E883-77, RRID:AB_725947), FOXO3A (Abcam, ab47285, RRID:AB_869819), Integrin beta 1 (Abcam, ab230291, Clone# 12G10), Jagged1 (Abcam, ab7771, RRID:AB_2280547), LAMP1 (Abcam, ab25630, Clone# H4A3, RRID:AB_470708), Nucleolin (Abcam, ab136649, Clone# 364-5), Podoplanin (Abcam, ab10288, Clone# 18H5, RRID:AB_297027), SC35 (Abcam, ab11826, Clone# SC-35, RRID:AB_298608), Smad3 (Abcam, ab52903, Clone# EP823Y, RRID:AB_882596), Human CD107b (BD Biosciences, ab555803, Clone# H4B4, RRID:AB_396137), Human Cyclin A (BD Biosciences, 611268, RRID:AB_398797), GOLPH4 (BosterBio, M10120-1, Clone# 21G09), CHD2 (Cell Engineering Corporation, CEC-040, Clone# 6D2), Cytokeratin18 (Cell Engineering Corporation, CEC-046, Clone# D2C7), Cytokeratin8 (Cell Engineering Corporation, CEC-045, Clone# 6F4F8), DHX9/RNA helicaseA (Cell Engineering Corporation, CEC-042, Clone# 8E3), Glis1 (Cell Engineering Corporation, CEC-054, Clone# 7-14), Histone H3.3 (Cell Engineering Corporation, CEC-008, Clone# 4H2D7), IGF-1R (Cell Engineering Corporation, CEC-061, Clone# 1B1), Importin 4 (cCell Engineering Corporation, CEC-023, Clone# 3C2), Importin alpha5/NPI-1 (Cell Engineering Corporation, CEC-021, Clone# 2D9), INI1/BAF47/hSNF5 (Cell Engineering Corporation, CEC-037, Clone# 2C2), Nup153 (Cell Engineering Corporation, CEC-030, Clone# R3G1), Nup62/p62 (Cell Engineering Corporation, CEC-028, Clone# 8A12), Nup98 (cCell Engineering Corporation, CEC-029, Clone# 2H10), Oct4/POU5F1 (Cell Engineering Corporation, CEC-053, Clone# 1C10), Prohibitin2 (Cell Engineering Corporation, CEC-052, Clone# 7F8E3), Septin9-PanC (Cell Engineering Corporation, CEC-047, Clone# 1G7), SUMO1 (Cell Engineering Corporation, CEC-043, Clone# 4D12), SUMO2/3 (Cell Engineering Corporation, CEC-044, Clone# 3H12), Atk (Cell Signaling Technology, 4691, Clone# C67E7, RRID:AB_915783), EphA2 (Cell Signaling Technology, 6997, Clone# D4A2, RRID:AB_10827743), Ki-67 (Cell Signaling Technology, 9129, Clone# D3B5, RRID:AB_2687446), MEK1/2 (Cell Signaling Technology, 4694, Clone# L38C12, RRID:AB_10695868), N-Cadherin (Cell Signaling Technology, 14215, Clone# 13A9, RRID:AB_2798427), NF-κB p65 (Cell Signaling Technology, 6956, Clone# L8F6, RRID:AB_10828935), Phospho-ATF-2 (Cell Signaling Technology, 61584, Clone# E6A8A), β-Catenin (Cell Signaling Technology, 84441, Clone# D10A8) CAPZA1 (GeneTex, GTX84737, Clone# 2G4, RRID:AB_10727309), CENPA (GeneTex, GTX13939, Clone# 3-19, RRID:AB_369391), p38 MAPK (GeneTex, GTX60771, Clone# 8G4D11), DOCK4 (LSBio (LifeSpan), LS-C105088, Clone# 3E7, RRID:AB_2094766), Human IL6ST / CD130 / gp130 (LifeSpan, LS-C828388, Clone# 8D4D2), H3.1 (Sigma-Aldrich, MABE952, Clone# 1D4F2, RRID:AB_2943330), 53BP1 (Novus, NB100-304, RRID:AB_350221), AKT1 (Novus, NBP1-69923, Clone# 17F6.B11), alpha Tubulin (Novus, NB100-690, Clone# DM1A, RRID:AB_2210209), Calmodulin (Novus, NB120-2860, Clone# 2DM, RRID:AB_788383), CD163 (Novus, NB110-40686, Clone# EDHu-1, RRID:AB_714951), CD31/PECAM-1 (Novus, NBP2-47785-0.1mg, Clone# C31.3 + C31.7 + C31.10, RRID:AB_2864381), CD68/SR-D1 (Novus, NBP2-34587, Clone# C68/684), CD81 (Novus, NBP1-44861, Clone# M38, RRID:AB_10008097), CS Citrate Synthase (Novus, NBP2-36771, Clone# CL2545), Cyclin B1 (Novus, NB100-2648, Clone# V92.1, RRID:AB_10003492), Cyclin D1 (Novus, NBP2-34816, Clone# SPM587), eEF1A1 (Novus, NBP2-59411, Clone# 23C11), EHD2 (Novus, H00030846-M01, Clone# 2D8, RRID:AB_2097332), EWSR1 (Novus, NBP1-92686, Clone# 5H7, RRID:AB_11028101), FAK (Novus, NBP2-67577, Clone# SC54-07), Ferritin Light Chain (Novus, NBP2-54574, Clone# FTL/1386), GFPT1 (Novus, NBP2-52472, Clone# 1F1A4), GRP78/HSPA5 (Novus, NBP2-12888, Clone# 1H11-1H7), Hsp47 (Novus, NBP2-22400, Clone# 1C4-1A6), HSP90 beta (Novus, NB110-61640, Clone# H9010, RRID:AB_963937), Integrin alpha 5/CD49e (Novus, NBP2-50146, Clone# SNAKA51), LAR/PTPRF (Novus, NBP2-42172, Clone# S165-38), Nucleobindin 1 (Novus, NBP2-01446, Clone# OTI1A5), p23/PTGES3 (Novus, NB110-96879, Clone# JJ6, RRID:AB_1260817), PBEF/Visfatin/NAMPT (Novus, NBP2-80036, Clone# OMNI379), Peroxiredoxin 2 (Novus, H00007001-M01, Clone# 4E10-2D2, RRID:AB_547278), PTBP1 (Novus, H00005725-M01, Clone# 3H8, RRID:AB_547430), RSK3 (Novus, NBP2-52555, Clone# 3C4C8), SAP155 (Novus, NBP2-75656, Clone# JB40-32), STING/TMEM173 (Novus, NBP2-24683, RRID:AB_2868483), Survivin (Novus, NBP2-80971, Clone# 1277A), TRAP alpha (Novus, NBP2-01026, Clone# OTI4C7), TUFM (Novus, NBP2-36753, Clone# CL2245), Vimentin (Novus, NBP1-97672, Clone# RV202, RRID:AB_2915945), Wnt-3a (Novus, NBP2-52588+B77, Clone# 1E6G4), YAP1 (Novus, NBP2-67467, Clone# SU33-06), Histone H2A.X (Acris Antibodies, AP01346PU-N, RRID:AB_1617147), PML (OriGene, TA326979), STAT3 (Acris Antibodies, AM00149PU-N, RRID:AB_1007496), ATF6 (Proteintech, 66563-1-Ig, Clone# 3B7E4, RRID:AB_2881924), Bcl-XL (Proteintech, 66020-1-Ig, Clone# 4C12A6, RRID:AB_11042315), BUB1(Proteintech, 13330-1-AP, RRID:AB_2814999), Emerin(Proteintech, 10351-1-AP, RRID:AB_2100056), Lamin B1 (Proteintech, 66095-1-Ig, Clone# 3C10G12, RRID:AB_11232208), Notch1(Proteintech, 10062-2-AP, RRID:AB_2153338), PAI-1 (Proteintech, 66261-1-Ig, Clone# 1H4A5, RRID:AB_2881648), PCM1(Proteintech, 19856-1-AP, RRID:AB_2878616), TGN46(Proteintech, 66477-1-Ig, Clone# 1F6D5, RRID:AB_2881843), Human CD155/PVR (R&D Systems, MAB25301, Clone# 300907, RRID:AB_2174021), Human ErbB2/Her2 (R&D Systems, MAB1129, Clone# 191924, RRID:AB_357477), Human HIPK2 (R&D systems, MAB9307-100, Clone# 493918), Human IL-8/CXCL8 (R&D Systems, MAB208, Clone# 6217, RRID:AB_2249110), Human Phospho-Src (R&D systems, Clone# 1246F, MAB2685), Human Syndecan-3 (R&D Systems, MAB35391, Clone# 374412, RRID:AB_2238777), Human TGF-beta (R&D Systems, AF3025, RRID:AB_2255910), Human/Mouse E-Cadherin (R&D Systems, AF748, RRID:AB_355568), Human/Mouse Frizzled-7 (R&D Systems, MAB1981-050, Clone# 151143, RRID:AB_357996), Human/Mouse/Rat GSK-3 beta (R&D Systems, MAB2506, Clone# 272536, RRID:AB_2115341), Human/Mouse/Rat SOD2/Mn-SOD (R&D Systems, MAB3419, Clone# 349810, RRID:AB_2191831), Myosin Heavy Chain (R&D Systems, MAB4470, Clone# MF20, RRID:AB_1293549), EZH2 (Rockland, 600-401-BC6, RRID:AB_2613052), ABCD3 (Santa Cruz Biotechnology, sc-514728, Clone# F-1), ATRX (Santa Cruz Biotechnology, sc-55584, Clone# D-5, RRID:AB_831012), beta Tubulin (Santa Cruz Biotechnology, sc-53140, Clone# 3F3-G2, RRID:AB_793543), caveolin-1 (Santa Cruz Biotechnology, sc-53564, Clone# 7C8, RRID:AB_628859), CD164 (Santa Cruz Biotechnology, sc-271179, Clone# H-4, RRID:AB_10613973), COPA (Santa Cruz Biotechnology, sc-398099, Clone# H-3), Exportin T (Santa Cruz Biotechnology, sc-514591, Clone# D-11), Filamin 1 (Santa Cruz Biotechnology, sc-71118, Clone# 3F180, RRID:AB_2231965), Fra1 (Santa Cruz Biotechnology, sc-28310, Clone# C-12, RRID:AB_627632), Histone Deacetylase 2 (Santa Cruz Biotechnology, sc-9959, Clone# C-8, RRID:AB_627704), HMG-I/HMG-Y (Santa Cruz Biotechnology, sc-393213, Clone# D-12), hnRNP C1/C2 (Santa Cruz Biotechnology, sc-32308, Clone# 4F4, RRID:AB_627731), IQGAP1 (Santa Cruz Biotechnology, sc-376021, Clone# C-9, RRID:AB_10988556), Ku86 (Santa Cruz Biotechnology, sc-515736, Clone# B-4, RRID:AB_2890940), MTA2 (Santa Cruz Biotechnology, sc-55566, Clone# F-9, RRID:AB_831564), NUMB (Santa Cruz Biotechnology, sc-136554, Clone# 48, RRID:AB_10611794), p-4E-BP1/2/3 (Santa Cruz Biotechnology, sc-271947, Clone# A-10, RRID:AB_10709575), p-Atm (Santa Cruz Biotechnology, sc-47739, Clone# 10H11.E12, RRID:AB_781524) p-ERK (Santa Cruz Biotechnology, sc-7383, Clone# E-4, RRID:AB_627545), p-Histone H2A.X (Santa Cruz Biotechnology, sc-517348, RRID:AB_2783871), p-MEK-3/6 (Santa Cruz Biotechnology, sc-8407, Clone# B-9, RRID:AB_627924), PSF (Santa Cruz Biotechnology, sc-101137, Clone# 39-1, RRID:AB_1129397), Ran (Santa Cruz Biotechnology, sc-271376, Clone# A-7, RRID:AB_10610890), SAP 14 (Santa Cruz Biotechnology, sc-514930, Clone# C-12), SPARC (Santa Cruz Biotechnology, sc-398419, Clone# D-2), SQSTM1/p62 (Santa Cruz Biotechnology, sc-28359, Clone# D-3, RRID:AB_628279), Tara (Santa Cruz Biotechnology, sc-377474, Clone# G-9), Tom20 (Santa Cruz Biotechnology, sc-17764, Clone# F-10, RRID:AB_628381), Transgelin (Santa Cruz Biotechnology, sc-53932, Clone# 6G6, RRID:AB_1129519), Transketolase (Santa Cruz Biotechnology, sc-390179, Clone# H-7, RRID:AB_2925185), UVRAG (Santa Cruz Biotechnology, sc-293268, Clone# 2E8), Waf1/Cip1/CDKN1A p21 (Santa Cruz Biotechnology, sc-817, Clone# 187, RRID:AB_628072), YB-1 (Santa Cruz Biotechnology, sc-398146, Clone# E-7), α-Actin (Santa Cruz Biotechnology, sc-32251, Clone# 1A4, RRID:AB_262054), APC (Sigma-Aldrich, WH0000324M1, Clone# 3E2, RRID:AB_1839711), LAMIN A (Sigma-Aldrich, L1293, RRID:AB_532254), Laminin-2 (Sigma-Aldrich, L0663, Clone# 4H8-2, RRID:AB_477153), phospho-p38 (Millipore, MABS64, Clone# 6E5.2, RRID:AB_10631874), ALDOA (Thermo Fisher Scientific, H00000226-M01, Clone# 3D9-6F3), Alpha-Smooth Muscle Actin (Thermo Fisher Scientific, 14-9760-82, Clone# 1A4,

RRID:AB_2572996), B-Raf (Thermo Fisher Scientific, 702187, Clone# 7H30L21, RRID:AB_2633065), CD44 (Thermo Fisher Scientific, MA4400, Clone# Hermes-1, RRID:AB_223517), CHD1 (Thermo Fisher Scientific, MA5-24306, Clone# 677616, RRID:AB_2610451), Clathrin Heavy Chain (Thermo Fisher Scientific, MA1-065, Clone# X22, RRID:AB_2083179), Cyclin E (Thermo Fisher Scientific, MA5-32349, Clone# SD20-24, RRID:AB_2809630), EpCAM (Thermo Fisher Scientific, MA1-10195, Clone# VU-1D9, RRID:AB_11153547), Fibronectin (Thermo Fisher Scientific, MIF2601, Clone# 3F12, RRID:AB_11154171), Filamin B (Thermo Fisher Scientific, MA5-27748, Clone# GT387, RRID:AB_2735183), FOXO3A (Thermo Fisher Scientific, 701847, Clone# 5H17L19, RRID:AB_2633032), FUBP1 (Thermo Fisher Scientific, MA5-32048, Clone# SY11-04, RRID:AB_2809342), Glutaminase C (Thermo Fisher Scientific, MA5-31538, Clone# GT3211, RRID:AB_2787166), GPIP137(Thermo Fisher Scientific, 703658, Clone# 6H17L10, RRID:AB_2784596), HES1 (Thermo Fisher Scientific, H00003280-M02, Clone# 3A3), IL-1 alpha (Thermo Fisher Scientific, MA5-23694, Clone# 4414, RRID:AB_2609800), IL-6 (Thermo Fisher Scientific, 701028, Clone# 4H16L21, RRID:AB_2532352), Ku70/Ku80 (Thermo Fisher ScientificMA5-12938, Clone# 162, RRID:AB_10980693), Laminin (Thermo Fisher Scientific, MA1-06100, Clone# A5, RRID:AB_559896), mTOR (Thermo Fisher Scientific, MA5-31505, Clone# GT649, RRID:AB_2787136), Nestin (Thermo Fisher Scientific, 14-9843-82, Clone# 10C2, RRID:AB_1548837), NPM1 (Thermo Fisher Scientific, 32-5200, Clone# FC-61991, RRID:AB_2533084), PAK1 (Thermo Fisher Scientific, H00005058-M02, Clone# 4D1), Phospho-EIF2S1 (Thermo Fisher Scientific, 701268, Clone# 10H21L20, RRID:AB_2532450), Phospho-JNK1/JNK2 (Thermo Fisher Scientific, 700031, Clone# D12H7L17, RRID:AB_2532273), PI3K p85 alpha (Thermo Fisher Scientific, MA5-17150, Clone# 6G10, RRID:AB_2538621), PKM2 (Thermo Fisher Scientific, 703707, Clone# 3H18L21, RRID:AB_2784601), PRDM14 (Thermo Fisher Scientific, MA5-24292, Clone# 647002, RRID:AB_2605535), RAD50 (Thermo Fisher Scientific, MA1-23269, Clone# 13B3, RRID:AB_560822), SIRT1 (Thermo Fisher Scientific, 703653, Clone# 2H9L18, RRID:AB_2784594), SMAD1/SMAD5 (Thermo Fisher Scientific, 700047, Clone# 31H14L11, RRID:AB_2532276), SR (Thermo Fisher Scientific, 33-9300, Clone# 16H3, RRID:AB_2533149), TEF1 (Thermo Fisher Scientific, MA5-27786, Clone# GT13112, RRID:AB_2735391), AffiniPure Fab Fragment Goat Anti-Rabbit IgG, Fc fragment specific (Jackson ImmunoResearch Labs, 111-007-008, RRID:AB_2632459), AffiniPure Fab Fragment Goat Anti-Mouse IgG1, Fcγ fragment specific (Jackson ImmunoResearch Labs, 115-007-185, RRID:AB_2632498), AffiniPure Fab Fragment Goat Anti-Mouse IgG2a, Fcγ fragment specific (Jackson ImmunoResearch Labs, 115-007-186, RRID:AB_2632499), AffiniPure Fab Fragment Goat Anti-Mouse IgG2b, Fcγ fragment specific (Jackson ImmunoResearch Labs, 115-007-187, RRID:AB_2632500), AffiniPure Fab Fragment Donkey Anti-Rat IgG (H+L) (Jackson ImmunoResearch Labs, 712-007-003, RRID:AB_2340634), AffiniPure Fab Fragment Bovine Anti-Goat IgG, Fc fragment specific (Jackson ImmunoResearch Labs, 805-007-008, RRID:AB_2632573), H3K14ac (Clone# 7G8, Karmodiya et al., BMC Genomics 2012, PMID: 22920947), H3K18ac (Clone# 20D2, http://kimura-lab.bio.titech.ac.jp), H3K23ac (Clone# 49D7, http://kimura-lab.bio.titech.ac.jp), H3K27ac (Clone# 9E2H10, Kimura et al., Cell Structure Func 2008, PMID: 18227620), H3K27me1 (Clone# 1B3, Hayashi-Takanaka et al., J Cell Sci 2020,PMID: 32576661), H3K27me3 (Clone# 1E7, Hayashi-Takanaka et al., Nucleic Acids Res 2011,PMID: 21576221), H3K36me2 (Clone# 2C3, Rechtsteiner et al., PLoS Genet 2010 ,PMID: 20824077), H3K36me3 (Clone# 13C9, Rechtsteiner et al., PLoS Genet 2010 ,PMID: 20824077), H3K4me1 (Clone# 19A5, Kimura et al., Cell Structure Func 2008, PMID: 18227620), H3K4me2 (Clone# 27A6, Kimura et al., Cell Structure Func 2008, PMID: 18227620), H3K4me3 (Clone# 16H10, Kimura et al., Cell Structure Func 2008, PMID: 18227620), H3K9ac (Clone# 19E5, Kimura et al., Cell Structure Func 2008, PMID: 18227620), H3K9me2 (Clone# 6D11, Hayashi-Takanaka et al., Nucleic Acids Res 2011, PMID: 21576221), H3K9me3 (Clone# 2F3, Chandra et al., Mol Cell 2012, PMID: 22795131), H3S10Ph (Hayashi-Tanaka et al., J.C.B 2009, PMID: 19995936), H3S28Ph (Hayashi-Tanaka et al., Plos One 2014, PMID: 25184362), H4K12ac (Clone# 50B3, Hayashi-Tanaka et al., Chromosome Res 2015, PMID: 26343042), H4K16ac (Clone# 1B2, Hayashi-Tanaka et al., Chromosome Res 2015, PMID: 26343042), H4K20me1 (Clone# 15F11, Hayashi-Tanaka et al., Chromosome Res 2015, PMID: 26343042), H4K20me2 (Clone# 2E2, Hayashi-Tanaka et al., Chromosome Res 2015, PMID: 26343042), H4K20me3 (Clone# 27F10, Hayashi-Tanaka et al., Chromosome Res 2015, PMID: 26343042), H4K5ac (Clone# 4A7, Hayashi-Tanaka et al., Chromosome Res 2015, PMID: 26343042), H4K8ac (Clone# 72A9, Hayashi-Tanaka et al., Chromosome Res 2015, PMID: 26343042), Pol II CTD (Clone# C13, Stasevich et al., Nature 2014, PMID: 25252976), Pol II Ser2Ph (Clone# Pc26, Stasevich et al., Nature 2014, PMID: 25252976), Pol II Ser5Ph (Clone# Pa57, Stasevich et al., Nature 2014, PMID: 25252976)

Validation

Anti-AHNAK used for IF, reactive to Human, was validated by the company (https://www.abcam.com/en-pt/products/primary-antibodies/ahnak-antibody-em-09-ab68556).
Anti-Annexin-2 used for IF, reactive to Human, was validated by the company (https://www.abcam.com/en-re/products/primary-antibodies/annexin-2-anxa2-antibody-d1-2745-ab269511).
Anti-Axl used for IF, reactive to Human, was validated by the company (https://www.abcam.com/en-ba/products/primary-antibodies/axl-antibody-mm0098-2n33-ab89224).
Anti-Calnexin used for IF, reactive to Human, was validated by the company (https://www.abcam.com/en-fi/products/primary-antibodies/calnexin-antibody-er-marker-ab22595).
Anti-Calreticulin used for IF, reactive to Human, was validated by the company (https://www.abcam.com/en-th/products/primary-antibodies/calreticulin-antibody-fmc-75-ab22683).
Anti-Cleaved Caspase-3 used for IF, reactive to Human, was validated by the company (https://www.abcam.com/en-kr/products/primary-antibodies/cleaved-caspase-3-antibody-e83-77-ab32042).
Anti-FOXO3A used for IF, reactive to Human, was validated by the company (https://www.abcam.com/en-pl/products/primary-antibodies/foxo3a-phospho-s253-antibody-ab47285).
Anti-Integrin beta 1 used for IF, reactive to Human, was validated by the company (https://www.abcam.com/en-it/products/primary-antibodies/integrin-beta-1-antibody-12g10-bsa-and-azide-free-ab230291).
Anti-Jagged1 used for IF, reactive to Human, was validated by the company (https://www.abcam.com/en-gh/products/primary-antibodies/jagged1-antibody-ab7771).
Anti-LAMP1 used for IF, reactive to Human, was validated by the company (https://www.abcam.com/en-it/products/primary-antibodies/lamp1-antibody-h4a3-ab25630#).
Anti-Nucleolin used for IF, reactive to Human, was validated by the company (https://www.abcam.com/en-cl/products/primary-antibodies/nucleolin-antibody-364-5-ab136649).
Anti-Podoplanin used for IF, reactive to Human, was validated by the company (https://www.abcam.com/en-at/products/primary-antibodies/podoplanin-antibody-18h5-bsa-and-azide-free-ab10288).
Anti-SC35 used for IF, reactive to Human, was validated by the company (https://www.abcam.com/en-mg/products/primary-antibodies/sc35-antibody-sc-35-nuclear-speckle-marker-ab11826).
Anti-Smad3 used for IF, reactive to Human, was validated by the company (https://www.abcam.com/en-pl/products/primary-antibodies/smad3-phospho-s423-s425-antibody-ep823y-ab52903).
Anti-CD107b used for IF, reactive to Human, was validated by the company (https://www.bdbiosciences.com/en-us/products/reagents/flow-cytometry-reagents/research-reagents/single-color-antibodies-ruo/purified-mouse-anti-human-cd107b.555803).

Anti-Cyclin A used for IF, reactive to Human, was validated by the company (https://www.bdbiosciences.com/en-us/products/reagents/microscopy-imaging-reagents/immunofluorescence-reagents/purified-mouse-anti-human-cyclin-a.611268).
Anti-GOLPH4 used for IF, reactive to Human, was validated by the company (https://www.bosterbio.com/anti-golph4-rabbit-monoclonal-antibody-m10120-1-boster.html). See also antibody pedia(https://www.antibodypedia.com/gene/952/GOLIM4/antibody/3853144/M10120).
Anti-CHD2 used for IF, reactive to Human, was validated by the company (https://store.cell-eng.com/products/anti-chd2-mab-6d2-cec-040).
Anti-Cytokeratin18 used for IF, reactive to Human, was validated by the company (https://store.cell-eng.com/products/anti-cytokeratin18-ck18-mab-d2c7-cec-046?_pos=1&_sid=d7c3c779a&_ss=r).
Anti-Cytokeratin8 used for IF, reactive to Human, was validated by the company (https://store.cell-eng.com/products/anti-cytokeratin8-ck8-mab-6f4f8-cec-045?_pos=1&_sid=129bb51cb&_ss=r).
Anti-DHX9/RNA helicaseA used for IF, reactive to Human, was validated by the company (https://store.cell-eng.com/products/anti-dhx9-rna-helicasea-mab-8e3-cec-042?_pos=1&_sid=324c1b297&_ss=r).
Anti-Glis1 used for IF, reactive to Human, was validated by the company (https://store.cell-eng.com/products/anti-glis1-mab-7-14-cec-054?_pos=1&_sid=f1638715a&_ss=r).
Anti-Histone H3.3 used for IF, reactive to Human, was validated by the company (https://store.cell-eng.com/products/anti-histone-h3-3-mabclone-no-4h2d7-4h2d7-cec-008?_pos=1&_sid=c1f5ea6d9&_ss=r).
Anti-IGF-1R used for IF, reactive to Human, was validated by the company (https://store.cell-eng.com/products/anti-igf-1r-mab-1b1-cec-061?_pos=1&_sid=4e601f09d&_ss=r).
Anti-Importin 4 used for IF, reactive to Human, was validated by the company (https://store.cell-eng.com/products/anti-importin-4-mab-3c2-cec-023?_pos=1&_sid=49abfad39&_ss=r).
Anti-Importin alpha5/NPI-1 used for IF, reactive to Human, was validated by the company (https://store.cell-eng.com/products/anti-importin-alpha5-npi-1-mab-2d9-cec-021?_pos=1&_sid=6fb71bd8c&_ss=r).
Anti-INI1/BAF47/hSNF5 used for IF, reactive to Human, was validated by the company (https://store.cell-eng.com/products/anti-ini1-baf47-hsnf5-mab-2c2-cec-037?_pos=1&_sid=c1a5fd744&_ss=r).
Anti-Nup153 used for IF, reactive to Human, was validated by the company (https://store.cell-eng.com/products/anti-nup153-mabclone-no-r3g1-r3g1-cec-030?_pos=1&_sid=144b67446&_ss=r).
Anti-Nup62/p62 used for IF, reactive to Human, was validated by the company (https://store.cell-eng.com/products/anti-nup62-p62-mabclone-no-8a12-8a12-cec-028?_pos=1&_sid=01e6d4705&_ss=r).
Anti-Nup98 used for IF, reactive to Human, was validated by the company (https://store.cell-eng.com/products/anti-nup98-mab-2h10-cec-029?_pos=1&_sid=c2e0d54d2&_ss=r).
Anti-Oct4/POU5F1 used for IF, reactive to Human, was validated by the company (https://store.cell-eng.com/products/anti-oct4-pou5f1-mab-1c10-cec-053?_pos=1&_sid=d6321a208&_ss=r).
Anti-Prohibitin2 used for IF, reactive to Human, was validated by the company (https://store.cell-eng.com/products/anti-prohibitin2-phb2-mab-7f8e3-cec-052?_pos=1&_sid=3c64c56f6&_ss=r).
Anti-Septin9-PanC used for IF, reactive to Human, was validated by the company (https://store.cell-eng.com/products/anti-septin9-panc-mab-1g7-cec-047?_pos=1&_sid=676a88ea6&_ss=r).
Anti-SUMO1 used for IF, reactive to Human, was validated by the company (https://store.cell-eng.com/products/anti-sumo1-mab-4d12-cec-043?_pos=1&_sid=295ff9457&_ss=r).
Anti-SUMO2/3 used for IF, reactive to Human, was validated by the company (https://store.cell-eng.com/products/anti-sumo2-3-mab-3h12-cec-044?_pos=1&_sid=c1dd13b2b&_ss=r).
Anti-Akt used for IF, reactive to Human, was validated by the company (https://www.cellsignal.jp/products/primary-antibodies/akt-pan-c67e7-rabbit-mab/4691).
Anti-EphA2 used for IF, reactive to Human, was validated by the company (https://www.cellsignal.jp/products/primary-antibodies/epha2-d4a2-xp-rabbit-mab/6997).
Anti-Ki-67 used for IF, reactive to Human, was validated by the company (https://www.cellsignal.jp/products/primary-antibodies/ki-67-d3b5-rabbit-mab/9129).
Anti-MEK1/2 used for IF, reactive to Human, was validated by the company (https://www.cellsignal.jp/products/primary-antibodies/mek1-2-l38c12-mouse-mab/4694).
Anti-N-Cadherin used for IF, reactive to Human, was validated by the company (https://www.cellsignal.jp/products/primary-antibodies/n-cadherin-13a9-mouse-mab/14215).
Anti-NF-κB p65 used for IF, reactive to Human, was validated by the company (https://www.cellsignal.com/products/primary-antibodies/nf-kb-p65-l8f6-mouse-mab/6956).
Anti-Phospho-ATF-2 used for IF, reactive to Human, was validated by the company (https://www.cellsignal.jp/products/primary-antibodies/phospho-atf-2-thr69-71-atf-7-thr51-53-e6a8a-rabbit-mab/61584).
Anti-β-Catenin used for IF, reactive to Human, was validated by the company (https://www.cellsignal.jp/products/primary-antibodies/b-catenin-d10a8-xp-174-rabbit-mab-bsa-and-azide-free/84441).
Anti-CAPZA1 used for IF, reactive to Human, was validated by the company (https://www.genetex.com/Product/Detail/CAPZA1-antibody-2G4/GTX84737).
Anti-CENPA used for IF, reactive to Human, was validated by the company (https://www.genetex.com/Product/Detail/CENPA-antibody-3-19/GTX13939).
Anti-p38 MAPK used for IF, reactive to Human, was validated by the company (https://www.genetex.com/Product/Detail/p38-MAPK-antibody-8G4D11/GTX60771).
Anti-DOCK4 used for IF, reactive to Human, was validated by the company (https://www.lsbio.com/antibodies/dock4-antibody-clone-3e7-elisa-if-immunofluorescence-wb-western-ls-c105088/106966).
Anti-Human IL6ST / CD130 / gp130 used for IF, reactive to Human, was validated by the company (https://www.lsbio.com/antibodies/il6st-antibody-cd130-antibody-gp130-antibody-clone-8d4d2-elisa-flow-icc-ihc-wb-western-ls-c828388/855510).
Anti-H3.1 used for IF, reactive to Human, was validated by the company (https://www.merckmillipore.com/JP/ja/product/Anti-Histone-H3.1-Antibody-clone-1D4F2,MM_NF-MABE952).
Anti-53BP1 used for IF, reactive to Human, was validated by the company (https://www.novusbio.com/products/53bp1-antibody_nb100-304).
Anti-pAKT1 used for IF, reactive to Human, was validated by the company (https://www.novusbio.com/products/akt1-

antibody-17f6b11_nbp1-69923).
Anti-alpha Tubulin used for IF, reactive to Human, was validated by the company (https://www.novusbio.com/products/alpha-tubulin-antibody-dm1a_nb100-690).
Anti-Calmodulin used for IF, reactive to Human, was validated by the company (https://www.novusbio.com/products/calmodulin-antibody-2d1_nb120-2860).
Anti-CD163 used for IF, reactive to Human, was validated by the company (https://www.novusbio.com/products/cd163-antibody-edhu-1_nb110-40686).
Anti-CD31/PECAM-1 used for IF, reactive to Human, was validated by the company (https://www.novusbio.com/products/cd31-pecam-1-antibody-c313-c317-c3110_nbp2-47785).
Anti-CD68/SR-D1 used for IF, reactive to Human, was validated by the company (https://www.novusbio.com/products/cd68-sr-d1-antibody-c68-684_nbp2-34587).
Anti-CD81 used for IF, reactive to Human, was validated by the company (https://www.novusbio.com/products/cd81-antibody-m38_nbp1-44861).
Anti-CS Citrate Synthase used for IF, reactive to Human, was validated by the company (https://www.novusbio.com/products/cs-citrate-synthase-antibody-cl2545_nbp2-36771).
Anti-Cyclin B1 used for IF, reactive to Human, was validated by the company (https://www.novusbio.com/products/cyclin-b1-antibody-v921_nb100-2648).
Anti-Cyclin D1 used for IF, reactive to Human, was validated by the company (https://www.novusbio.com/products/cyclin-d1-antibody-spm587_nbp2-34816).
Anti-eEF1A1 used for IF, reactive to Human, was validated by the company (https://www.novusbio.com/products/eef1a1-antibody-23c11_nbp2-59411).
Anti-EHD2 used for IF, reactive to Human, was validated by the company (https://www.novusbio.com/products/ehd2-antibody-2d8_h00030846-m01).
Anti-EWSR1 used for IF, reactive to Human, was validated by the company (https://www.novusbio.com/products/ewsr1-antibody-5h7_nbp1-92686).
Anti-pFAK used for IF, reactive to Human, was validated by the company ()https://www.novusbio.com/products/fak-antibody-sc54-07_nbp2-67577.
Anti-Ferritin Light Chain used for IF, reactive to Human, was validated by the company (https://www.novusbio.com/products/ferritin-light-chain-antibody-ftl-1386_nbp2-54574).
Anti-GFPT1 used for IF, reactive to Human, was validated by the company (https://www.novusbio.com/products/gfpt1-antibody-1f1a4_nbp2-52472).
Anti-GRP78/HSPA5 used for IF, reactive to Human, was validated by the company (https://www.novusbio.com/products/grp78-hspa5-antibody-1h11-1h7_nbp2-12888).
Anti-Hsp47 used for IF, reactive to Human, was validated by the company (https://www.novusbio.com/products/hsp47-antibody-1c4-1a6_nbp2-22400).
Anti-HSP90 beta used for IF, reactive to Human, was validated by the company (https://www.novusbio.com/products/hsp90-beta-antibody-h9010_nb110-61640).
Anti-Integrin alpha 5/CD49e used for IF, reactive to Human, was validated by the company (https://www.novusbio.com/products/integrin-alpha-5-cd49e-antibody-snaka51_nbp2-50146).
Anti-LAR/PTPRF used for IF, reactive to Human, was validated by the company (https://www.novusbio.com/products/lar-ptprf-antibody-s165-38_nbp2-42172).
Anti-Nucleobindin 1 used for IF, reactive to Human, was validated by the company (https://www.novusbio.com/products/nucleobindin-1-antibody-oti1a5_nbp2-01446).
Anti-p23/PTGES3 used for IF, reactive to Human, was validated by the company (https://www.novusbio.com/products/p23-ptges3-antibody-jj6_nb110-96879).
Anti-PBEF/Visfatin/NAMPT used for IF, reactive to Human, was validated by the company (https://www.novusbio.com/products/pbef-visfatin-nampt-antibody-omni379_nbp2-80036).
Anti-Peroxiredoxin 2 used for IF, reactive to Human, was validated by the company (https://www.novusbio.com/products/peroxiredoxin-2-antibody-4e10-2d2_h00007001-m01).
Anti-PTBP1 used for IF, reactive to Human, was validated by the company (https://www.novusbio.com/products/ptbp1-antibody-3h8_h00005725-m01).
Anti-RSK3 used for IF, reactive to Human, was validated by the company (https://www.novusbio.com/products/rsk3-antibody-3c4c8_nbp2-52555).
Anti-SAP155 used for IF, reactive to Human, was validated by the company (https://www.novusbio.com/products/sap155-antibody-jb40-32_nbp2-75656).
Anti-STING/TMEM173 used for IF, reactive to Human, was validated by the company (https://www.novusbio.com/products/sting-tmem173-antibody_nbp2-24683).
Anti-Survivin used for IF, reactive to Human, was validated by the company (https://www.novusbio.com/products/survivin-antibody-1277a_nbp2-80971).
Anti-TRAP alpha used for IF, reactive to Human, was validated by the company (https://www.novusbio.com/products/trap-alpha-antibody-oti4c7_nbp2-01026).
Anti-TUFM used for IF, reactive to Human, was validated by the company (https://www.novusbio.com/products/tufm-antibody-cl2245_nbp2-36753).
Anti-Vimentin used for IF, reactive to Human, was validated by the company (https://www.novusbio.com/products/vimentin-antibody-rv202_nbp1-97672).
Anti-Wnt-3a used for IF, reactive to Human, was validated by the company (https://www.novusbio.com/products/wnt-3a-antibody-1e6g4_nbp2-52588).
Anti-YAP1 used for IF, reactive to Human, was validated by the company (https://www.novusbio.com/products/yap1-antibody-su33-06_nbp2-67467).
Anti-Histone H2A.X used for IF, reactive to Human, was validated by the company (https://www.antibodyregistry.org/AB_1617150).
Anti-PML used for IF, reactive to Human, was validated by the company (https://www.origene.com/catalog/antibodies/primary-antibodies/ta326979/pml-protein-pml-rabbit-polyclonal-antibody).

Anti-STAT3 used for IF, reactive to Human, was validated by the company (https://www.origene.com/catalog/antibodies/primary-antibodies/am00149pu-n/stat3-pser727-incl-pos-control-mouse-monoclonal-antibody-clone-id-23g5).
Anti-ATF6 used for IF, reactive to Human, was validated by the company (https://www.ptglab.co.jp/products/ATF6-Antibody-66563-1-Ig.htm).
Anti-Bcl-XL used for IF, reactive to Human, was validated by the company (https://www.ptglab.co.jp/products/Bcl-xL-Antibody-66020-1-Ig.htm).
Anti-BUB1 used for IF, reactive to Human, was validated by the company (https://www.ptglab.co.jp/products/BUB1-Antibody-13330-1-AP.htm).
Anti-Emerin used for IF, reactive to Human, was validated by the company (https://www.ptglab.co.jp/products/EMD-Antibody-10351-1-AP.htm).
Anti-Lamin B1 used for IF, reactive to Human, was validated by the company (https://www.ptglab.co.jp/products/LMNB1-Antibody-66095-1-Ig.htm).
Anti-Notch1 used for IF, reactive to Human, was validated by the company (https://www.ptglab.co.jp/products/Notch1-Antibody-10062-2-AP.htm).
Anti-PAI-1 used for IF, reactive to Human, was validated by the company (https://www.ptglab.co.jp/products/PAI-1-Antibody-66261-1-Ig.htm).
Anti-PCM1 used for IF, reactive to Human, was validated by the company (https://www.ptglab.co.jp/products/PCM1-Antibody-19856-1-AP.htm).
Anti-TGN46 used for IF, reactive to Human, was validated by the company (https://www.ptglab.co.jp/products/TGOLN2,TGN46-Antibody-66477-1-Ig.htm).
Anti-Human CD155/PVR used for IF, reactive to Human, was validated by the company (https://www.rndsystems.com/products/human-cd155-pvr-antibody-300907_mab25301).
Anti-Human ErbB2/Her2 used for IF, reactive to Human, was validated by the company (https://www.rndsystems.com/products/human-erbb2-her2-antibody-191924_mab1129).
Anti-Human HIPK2 used for IF, reactive to Human, was validated by the company (https://www.rndsystems.com/products/human-hipk2-antibody-493918_mab9307).
Anti-Human IL-8/CXCL8 used for IF, reactive to Human, was validated by the company (https://www.rndsystems.com/products/human-il-8-cxcl8-antibody-6217_mab208).
Anti-Human Phospho-Src used for IF, reactive to Human, was validated by the company (https://resources.rndsystems.com/pdfs/datasheets/mab2685.pdf).
Anti-Human Syndecan-3 used for IF, reactive to Human, was validated by the company (https://www.rndsystems.com/products/human-syndecan-3-antibody-374412_mab35391).
Anti-Human TGF-beta used for IF, reactive to Human, was validated by the company (https://www.rndsystems.com/products/human-tgf-beta-ri-alk-5-antibody_af3025).
Anti-Human/Mouse E-Cadherin used for IF, reactive to Human, was validated by the company (https://www.rndsystems.com/products/human-mouse-e-cadherin-antibody_af748).
Anti-Human/Mouse Frizzled-7 used for IF, reactive to Human, was validated by the company (https://www.rndsystems.com/products/human-mouse-frizzled-7-antibody-151143_mab1981).
Anti-Human/Mouse/Rat GSK-3 beta used for IF, reactive to Human, was validated by the company (https://www.rndsystems.com/products/human-mouse-rat-gsk-3beta-antibody-272536_mab2506).
Anti-Human/Mouse/Rat SOD2/Mn-SOD used for IF, reactive to Human, was validated by the company (https://www.rndsystems.com/products/human-mouse-rat-sod2-mn-sod-antibody-349810_mab3419).
Anti-Myosin Heavy Chain used for IF, reactive to Human, was validated by the company (https://www.rndsystems.com/products/myosin-heavy-chain-antibody-mf20_mab4470).
Anti-EZH2 used for IF, reactive to Human, was validated by the company (https://www.rockland.com/categories/primary-antibodies/ezh2-antibody-600-401-BC6/).
Anti-ABCD3 used for IF, reactive to Human, was validated by the company (https://datasheets.scbt.com/sc-514728.pdf).
Anti-ATRX used for IF, reactive to Human, was validated by the company (https://datasheets.scbt.com/sc-55584.pdf).
Anti-beta Tubulin used for IF, reactive to Human, was validated by the company (https://datasheets.scbt.com/sc-53140.pdf).
Anti-caveolin-1 used for IF, reactive to Human, was validated by the company (https://datasheets.scbt.com/sc-53564.pdf).
Anti-CD164 used for IF, reactive to Human, was validated by the company (https://datasheets.scbt.com/sc-271179.pdf).
Anti-COPA used for IF, reactive to Human, was validated by the company (https://datasheets.scbt.com/sc-398099.pdf).
Anti-Exportin T used for IF, reactive to Human, was validated by the company (https://datasheets.scbt.com/sc-514591.pdf).
Anti-Filamin 1 used for IF, reactive to Human, was validated by the company (https://datasheets.scbt.com/sc-71118.pdf).
Anti-Fra1 used for IF, reactive to Human, was validated by the company (https://datasheets.scbt.com/sc-28310.pdf).
Anti-Histone Deacetylase 2 used for IF, reactive to Human, was validated by the company (https://datasheets.scbt.com/sc-9959.pdf).
Anti-HMG-I/HMG-Y used for IF, reactive to Human, was validated by the company (https://datasheets.scbt.com/sc-393213.pdf).
Anti-hnRNP C1/C2 used for IF, reactive to Human, was validated by the company (https://datasheets.scbt.com/sc-32308.pdf).
Anti-IQGAP1 used for IF, reactive to Human, was validated by the company (https://datasheets.scbt.com/sc-376021.pdf).
Anti-Ku86 used for IF, reactive to Human, was validated by the company (https://datasheets.scbt.com/sc-515736.pdf).
Anti-MTA2 used for IF, reactive to Human, was validated by the company (https://datasheets.scbt.com/sc-55566.pdf).
Anti-NUMB used for IF, reactive to Human, was validated by the company (https://datasheets.scbt.com/sc-136554.pdf).
Anti-p-4E-BP1/2/3 used for IF, reactive to Human, was validated by the company (https://datasheets.scbt.com/sc-271947.pdf).
Anti-p-Atm used for IF, reactive to Human, was validated by the company (https://datasheets.scbt.com/sc-47739.pdf).
Anti-p-ERK used for IF, reactive to Human, was validated by the company (https://datasheets.scbt.com/sc-7383.pdf).
Anti-p-Histone H2A.X used for IF, reactive to Human, was validated by the company (https://datasheets.scbt.com/sc-517348.pdf).
Anti-p-MEK-3/6 used for IF, reactive to Human, was validated by the company (https://www.scbt.com/ja/p/p-mek-3-6-antibody-b-9).
Anti-PSF used for IF, reactive to Human, was validated by the company (https://datasheets.scbt.com/sc-101137.pdf).
Anti-Ran used for IF, reactive to Human, was validated by the company (https://datasheets.scbt.com/sc-271376.pdf).
Anti-SAP 14 used for IF, reactive to Human, was validated by the company (https://datasheets.scbt.com/sc-514930.pdf).
Anti-SPARC used for IF, reactive to Human, was validated by the company (https://www.scbt.com/ja/p/sparc-antibody-d-2).

Anti-SQSTM1/p62 used for IF, reactive to Human, was validated by the company (https://datasheets.scbt.com/sc-28359.pdf).
Anti-Tara used for IF, reactive to Human, was validated by the company (https://www.scbt.com/ja/p/tara-antibody-g-9).
Anti-Tom20 used for IF, reactive to Human, was validated by the company (https://datasheets.scbt.com/sc-17764.pdf).
Anti-Transgelin used for IF, reactive to Human, was validated by the company (https://datasheets.scbt.com/sc-53932.pdf).
Anti-Transketolase used for IF, reactive to Human, was validated by the company (https://datasheets.scbt.com/sc-390179.pdf).
Anti-UVRAG used for IF, reactive to Human, was validated by the company (https://datasheets.scbt.com/sc-293268.pdf).
Anti-Waf1/Cip1/CDKN1A p21 used for IF, reactive to Human, was validated by the company (https://datasheets.scbt.com/sc-817.pdf).
Anti-YB-1 used for IF, reactive to Human, was validated by the company (https://datasheets.scbt.com/sc-398146.pdf).
Anti-α-Actin used for IF, reactive to Human, was validated by the company (https://datasheets.scbt.com/sc-32251.pdf).
Anti-APC used for IF, reactive to Human, was validated by the company (https://www.sigmaaldrich.com/JP/ja/product/sigma/wh0000324m1).
Anti-LAMIN A used for IF, reactive to Human, was validated by the company (https://www.sigmaaldrich.com/JP/ja/product/sigma/l1293).
Anti-Laminin-2 used for IF, reactive to Human, was validated by the company (https://www.sigmaaldrich.com/JP/ja/product/sigma/l0663).
Anti-phospho-p38 used for IF, reactive to Human, was validated by the company (https://www.merckmillipore.com/JP/ja/product/Anti-phospho-p38-Thr180-Tyr182-Antibody-clone-6E5.2,MM_NF-MABS64).
Anti-ALDOA used for IF, reactive to Human, was validated by the company (https://www.thermofisher.com/antibody/product/ALDOA-Antibody-clone-3D9-6F3-Monoclonal/H00000226-M01).
Anti-Alpha-Smooth Muscle Actin used for IF, reactive to Human, was validated by the company (https://www.thermofisher.com/antibody/product/Alpha-Smooth-Muscle-Actin-Antibody-clone-1A4-Monoclonal/14-9760-82).
Anti-B-Raf used for IF, reactive to Human, was validated by the company (https://www.thermofisher.com/antibody/product/B-Raf-Antibody-clone-7H30L21-Recombinant-Monoclonal/702187).
Anti-CD44 used for IF, reactive to Human, was validated by the company (https://www.thermofisher.com/antibody/product/CD44-Chimeric-Antibody-clone-Hermes-3-Recombinant-Monoclonal/MA5-48018?ef_id=Cj0KCQjwq86wBhDiARIsAJhuphnYznLsDpB9PfAKAJou-UktJwP23oLisiorjRJBy87F9ByprODnQOUaAq_6EALw_wcB:G:s&s_kwcid=AL!3652!3!459737518508!!!g!!!10950825775!106531320406&cid=bid_pca_aup_r01_co_cp1359_pjt0000_bid00000_0se_gaw_dy_pur_con&gad_source=1&gclid=Cj0KCQjwq86wBhDiARIsAJhuphnYznLsDpB9PfAKAJou-UktJwP23oLisiorjRJBy87F9ByprODnQOUaAq_6EALw_wcB).
Anti-CHD1 used for IF, reactive to Human, was validated by the company (https://www.thermofisher.com/antibody/product/CHD1-Antibody-clone-677616-Monoclonal/MA5-24306).
Anti-Clathrin used for IF, reactive to Human, was validated by the company (https://www.thermofisher.com/antibody/product/Clathrin-Heavy-Chain-Antibody-clone-X22-Monoclonal/MA1-065).
Anti-Cyclin E used for IF, reactive to Human, was validated by the company (https://www.thermofisher.com/antibody/product/Cyclin-E-Antibody-clone-SD20-24-Recombinant-Monoclonal/MA5-32349).
Anti-EpCAM used for IF, reactive to Human, was validated by the company (https://www.thermofisher.com/antibody/product/EpCAM-Antibody-clone-VU-1D9-Monoclonal/MA1-10195).
Anti-Fibronectin used for IF, reactive to Human, was validated by the company (https://www.thermofisher.com/antibody/product/Fibronectin-Antibody-clone-3F12-Monoclonal/MIF2601).
Anti-Filamin B used for IF, reactive to Human, was validated by the company (https://www.thermofisher.com/antibody/product/Filamin-B-Antibody-clone-GT387-Monoclonal/MA5-27748).
Anti-FOXO3A used for IF, reactive to Human, was validated by the company (https://www.thermofisher.com/antibody/product/FOXO3A-Antibody-clone-5H17L19-Recombinant-Monoclonal/701847).
Anti-FUBP1 used for IF, reactive to Human, was validated by the company (https://www.thermofisher.com/antibody/product/FUBP1-Antibody-clone-SY11-04-Recombinant-Monoclonal/MA5-32048).
Anti-Glutaminase C used for IF, reactive to Human, was validated by the company (https://www.thermofisher.com/antibody/product/Glutaminase-C-GAC-Antibody-clone-GT3211-Monoclonal/MA5-31538).
Anti-GPIP137 used for IF, reactive to Human, was validated by the company (https://www.thermofisher.com/antibody/product/GPIP137-Antibody-clone-6H17L10-Recombinant-Monoclonal/703658).
Anti-HES1 used for IF, reactive to Human, was validated by the company (https://www.thermofisher.com/antibody/product/HES1-Antibody-clone-3A3-Monoclonal/H00003280-M02).
Anti-IL-1 alpha used for IF, reactive to Human, was validated by the company (https://www.thermofisher.com/antibody/product/IL-1-alpha-Antibody-clone-4414-Monoclonal/MA5-23694).
Anti-IL-6 used for IF, reactive to Human, was validated by the company (https://www.thermofisher.com/antibody/product/IL-6-Antibody-clone-4H16L21-Recombinant-Monoclonal/701028).
Anti-Ku70/Ku80 used for IF, reactive to Human, was validated by the company (https://rnaidesigner.qa.thermofisher.com/antibody/product/Ku70-Ku80-Antibody-clone-162-Monoclonal/MA5-12938).
Anti-Laminin used for IF, reactive to Human, was validated by the company (https://www.googleadservices.com/pagead/aclk?sa=L&ai=DChcSEwiKoJGrt7SFAxXIzBYFHblxC3oYABAAGgJ0bA&ae=2&gclid=Cj0KCQjwq86wBhDiARIsAJhuphmMmQHiT9P9w1bKy_WmSgYSEqybWGRF5Aq8qxyksEim_NzGOboVkZcaAjHcEALw_wcB&ohost=www.google.com&cid=CAESVeD2oikNNRraPElqA7OrkpWXrNORs75syTwgEcT6ryVltU10XNJa1xL0i85ajOK0xrqtwqEyD8LLGypF6XVg6xpbJh61Gk6kAda6_Ewpu_HHPCopBEU&sig=AOD64_02DdZqF6dWZNhKKiB-7CVqd-tbGA&q&adurl&ved=2ahUKEwjmzourt7SFAxWaiFYBHduRAg0Q0Qx6BAgIEAE&nis=8&dct=1).
Anti-mTOR used for IF, reactive to Human, was validated by the company (https://www.thermofisher.com/antibody/product/mTOR-Antibody-clone-GT649-Monoclonal/MA5-31505).
Anti-Nestin used for IF, reactive to Human, was validated by the company (https://www.thermofisher.com/antibody/product/Nestin-Antibody-clone-10C2-Monoclonal/14-9843-82).
Anti-NPM1 used for IF, reactive to Human, was validated by the company (https://www.thermofisher.com/antibody/product/NPM1-Antibody-clone-FC-61991-Monoclonal/32-5200).
Anti-PAK1 used for IF, reactive to Human, was validated by the company (https://www.thermofisher.com/antibody/product/PAK1-Antibody-clone-4D1-Monoclonal/H00005058-M02).
Anti-Phospho-EIF2S1 used for IF, reactive to Human, was validated by the company (https://www.thermofisher.com/antibody/

product/Phospho-EIF2S1-Ser51-Antibody-clone-10H21L20-Recombinant-Monoclonal/701268).
Anti-Phospho-JNK1/JNK2 used for IF, reactive to Human, was validated by the company (https://www.thermofisher.com/antibody/product/Phospho-JNK1-JNK2-Thr183-Tyr185-Antibody-clone-D12H7L17-Recombinant-Monoclonal/700031).
Anti-PI3K p85 alpha used for IF, reactive to Human, was validated by the company (https://www.thermofisher.com/antibody/product/PI3K-p85-alpha-Antibody-clone-6G10-Monoclonal/MA5-17150).
Anti-PKM2 used for IF, reactive to Human, was validated by the company (https://www.thermofisher.com/antibody/product/PKM2-Antibody-clone-3H18L21-Recombinant-Monoclonal/703707).
Anti-PRDM14 used for IF, reactive to Human, was validated by the company (https://www.thermofisher.com/antibody/product/PRDM14-Antibody-clone-647002-Monoclonal/MA5-24292).
Anti-RAD50 used for IF, reactive to Human, was validated by the company (https://www.thermofisher.com/antibody/product/RAD50-Antibody-clone-13B3-Monoclonal/MA1-23269).
Anti-SIRT1 used for IF, reactive to Human, was validated by the company (https://rnaidesigner.thermofisher.com/antibody/product/SIRT1-Antibody-clone-2H9L18-Recombinant-Monoclonal/703653).
Anti-pSMAD1/SMAD5 used for IF, reactive to Human, was validated by the company (https://rnaidesigner.thermofisher.com/antibody/product/Phospho-SMAD1-SMAD5-Ser463-Ser465-Antibody-clone-31H14L11-Recombinant-Monoclonal/700047).
Anti-SR used for IF, reactive to Human, was validated by the company (https://www.thermofisher.com/antibody/product/SR-Antibody-clone-16H3-16H3E8-Monoclonal/33-9300).
Anti-TEF1 used for IF, reactive to Human, was validated by the company (https://www.thermofisher.com/antibody/product/TEF1-Antibody-clone-GT13112-Monoclonal/MA5-27786).
Anti-H3K14ac used for IF, reactive to Human, was generated and validated as previously described [PMID: 22920947].
Anti-H3K18ac and H3K23ac used for IF, reactive to Human, were generated and validated in the lab[http://kimura-lab.bio.titech.ac.jp]
Anti-H3K27ac used for IF, reactive to Human, was generated and validated as previously described [PMID: 18227620]
Anti-H3K27me1 used for IF, reactive to Human, was generated and validated as previously described [PMID: 32576661]
Anti-H3K27me3 used for IF, reactive to Human, was generated and validated as previously described [PMID: 21576221]
Anti-H3K36me2 and H3K36me3 used for IF, reactive to Human, were generated and validated as previously described [PMID: 20824077]
Anti-H3K4me1, H3K4me2, H3K4me3 and H3K9ac used for IF, reactive to Human, were generated and validated as previously described [PMID: 18227620].
Anti-H3K9me2 used for IF, reactive to Human, was generated and validated as previously described [PMID: 21576221].
Anti-H3K9me3 used for IF, reactive to Human, was generated and validated as previously described [PMID: 22795131].
Anti-H3S10Ph used for IF, reactive to Human, was generated and validated as previously described [PMID: 19995936].
Anti-H3S28Ph used for IF, reactive to Human, was generated and validated as previously described [PMID: 25184362].
Anti-H4K12ac, used for IF, reactive to Human, H4K16ac, H4K20me1, H4K20me2, H4K20me3, H4K5ac, and H4K8ac were generated and validated as previously described [PMID: 26343042].
Anti-Pol II CTD, Pol II Ser2Ph, and Pol II Ser5Ph used for IF, reactive to Human, were generated and validated as previously described [PMID: 25252976].

Eukaryotic cell lines

Policy information about cell lines and Sex and Gender in Research

Cell line source(s)	A549 (ATCC)<br>IMR90(ATCC)<br>IMR90 ER:Ras cells established in Young et al., 2009 study [PMID:19279323] was used.
Authentication	No authentication was performed by the authors of this manuscript.
Mycoplasma contamination	Cells were regularly tested for mycoplasma contamination and always found to be negative.
Commonly misidentified lines<br>(See ICLAC register)	No commonly misidentified lines are used.

Animals and other research organisms

Policy information about studies involving animals; ARRIVE guidelines recommended for reporting animal research, and Sex and Gender in Research

Laboratory animals	C57BL/6N mouse (17 weeks) was used. Purchased mouse was immediately used to prepare tissue-section.
Wild animals	No wild animals were used in the study.
Reporting on sex	Male
Field-collected samples	No field samples.
Ethics oversight	All animal procedures were conducted in accordance with the Guidelines for the Care and Use of Laboratory Animals and were approved by the Institutional Animal Care and Use Committee (IACUC) at Kyushu University (Approved ID: A23-288-1).

Note that full information on the approval of the study protocol must also be provided in the manuscript.

Plants

Seed stocks	*Report on the source of all seed stocks or other plant material used. If applicable, state the seed stock centre and catalogue number. If plant specimens were collected from the field, describe the collection location, date and sampling procedures.*
Novel plant genotypes	*Describe the methods by which all novel plant genotypes were produced. This includes those generated by transgenic approaches, gene editing, chemical/radiation-based mutagenesis and hybridization. For transgenic lines, describe the transformation method, the number of independent lines analyzed and the generation upon which experiments were performed. For gene-edited lines, describe the editor used, the endogenous sequence targeted for editing, the targeting guide RNA sequence (if applicable) and how the editor was applied.*
Authentication	*Describe any authentication procedures for each seed stock used or novel genotype generated. Describe any experiments used to assess the effect of a mutation and, where applicable, how potential secondary effects (e.g. second site T-DNA insertions, mosiacism, off-target gene editing) were examined.*
