# Supplementary material for: Precise immunofluorescence canceling for highly multiplexed imaging to capture specific cell states
Source: Nat Commun. 2024 May 8;15:3657. doi: 10.1038/s41467-024-47989-9 (PMC11078938; doi:10.1038/s41467-024-47989-9)
Supplement: Supplementary file 4 — Description of Additional Supplementary Files [file 41467_2024_47989_MOESM4_ESM.pdf]

Title: Supplementary Data 1

Description: List of antibodies used in this study.
